# Gender differentials on productivity of rice farmers in south western Nigeria: An Oaxaca-Blinder decomposition approach

**DOI:** 10.1016/j.heliyon.2023.e22724

**Published:** 2023-11-22

**Authors:** Temitope O. Ojo, Lloyd J.S. Baiyegunhi

**Affiliations:** a Department of Agricultural Economics, Obafemi Awolowo University, Ile-Ife, Nigeria; b Disaster Management Training and Education Centre for Africa at the University of the Free State, P.O Box 339, Bloemfontein, 9300, South Africa; c Department of Agricultural Economics, University of KwaZulu-Natal, South Africa

**Keywords:** Gender, Rice productivity, Oaxaca-Blinder approach, Exogenous switching treatment regression Nigeria

## Abstract

Gender differences in productivity are one of the major obstacles impeding the development of agriculture in Africa and Nigerian particularly. With the Oaxaca-Blinder (OB) and exogenous switching treatment regression (ESTER) models, this study investigates the causes of the productivity differences among 360 sampled rice farmers in Nigeria as well as gender inequality in agricultural productivity. The findings showed that there is an inequalities between men and women, which contributes to a gender productivity gap of almost 29 % in favour of men. As a result, plots managed by women are 29 % less productive than plots handled by men. The analysis of the factors influencing gender variations in production reveals that the endowment component, which accounts for 15 % of the productivity gap, is significantly influenced by marital status, education, farm size, and access to market information. Similarly, the ESTER results show that the rice yield of FHHs would have decreased by 25.41 kg/ha (a 1.02 % reduction) if they had been assigned the same returns to the observed features of MHHs. This difference is significant at the 1 % level. Thus, the findings imply that the FHHs are not at a yield disadvantage when compared to the MHHs. Therefore, it can be said that there are gender productivity disparities in the Nigerian agricultural industry. As such, policy interventions aimed at empowering women must take these disparities into consideration as well as the causes that contribute to them. Overall, the results demonstrate that although policymakers and their development partners can use improved technologies to increase MHH and FHH yields, reducing the difference in market linkages is necessary to close the gender gap in rice productivity and provide FHHs with equal access to the market.

## 1 Introduction

Risks to agricultural productivity are most prevalent in underdeveloped countries. Although the primary cause and consequences of these hazards may vary throughout countries, the majority of farmers in these countries face them[1,2]. According to Ref. [3]; and [4]; production activities of agriculture in Africa are primarily more susceptible to climate change as compared to other socioeconomic and institutional factors. Sub-Saharan Africa's (SSA) smallholder farmers are particularly susceptible to production hazards because of rising rates of poverty, a lack of technological innovation, and an excessive reliance on rain-fed agriculture [5,6]. People in the aforementioned regions continue to rely heavily on the agricultural production of risky natural resources, such as rice, maize, and millet, as well as other food crops[7,8].

Almost 50 % of global population depends on rice as a staple food, positioning it as one of the most significant food crops[9]. By2025, rice production will need to double in order to meet dietary demands and keep up with the world's rapid population growth[10,11]. In the SSA countries, where smallholder farming predominates, rice productivity is typically low. For female farmers, the productivity gap is even more pronounced. Research has consistently shown that women experience a 20 %–30 % productivity gap in agriculture, which poses a significant obstacle to the growth of the agricultural industry in this area.

According to Ref. [12]; one of the most persistent gender myths is that women make up 70 % of the world's disadvantaged. However, research shows that women, including girls, experience disadvantages across the globe with regard to the development of their human capital, physical capital, including assets and land, and productivity gaps with respect to men, and voice in their homes and communities[13,14]. According to Ref. [15]; women face additional disadvantages due to their exclusion from decision-making processes in certain agricultural policies and programs that aim to enhance food security and production within the nation. Women's involvement in agricultural decision-making is influenced by a number of factors, including the socioeconomic status of farmers[16]. Women play a crucial role in the production process, so their marginalization or lack of participation in decision-making has an impact on the creation of policies that effectively address the issues faced by farmers.

[17] stated that there were significant variations in agricultural productivity between genders throughout Sub-Saharan Africa (SSA), ranging from 4 % to 40 %. These variations were primarily caused by variations in the application of agricultural inputs, tenure security and associated investments in land and advanced technologies, market and credit accessibility, institutional and cultural limitations impacting intra-household allotment of farm/plot supervision and marketing responsibilities. The gender gap prevents rice farmers from receiving the full benefits of the resources used in their production, which lowers the ratio of self-sufficiency and decreases per capita food production[18].

Information regarding gender differences in smallholder rice farmers' productivity is therefore desperately needed [19]. claim that a significant knowledge of the agricultural gender study in the context of developing nations can be gained from the discussion on gender roles and needs, management and access capacities, and decision over agricultural productivity. Gender is impacted differently by various gender roles and activities, which also have varying effects on the environment. In addition to cultural, political, and socio-demographic features[20,21], the inefficient intra-household allocation can be explained by the disparate ways in which men and women use productive inputs [22]. Due to their participation in and reliance on livelihood activities that directly depend on the natural environment, poor women are typically the ones who suffer the most from the effects of growing environmental degradation and depletion of natural resources. Being a significant crop, rice rose to prominence in a number of Nigerian studies.

While some studies concentrated on resource-use efficiency [23] and technical efficiency (Ojo and Baiyegunhi, 2021), others considered the adoption of improved rice varieties [24], the impact of credit demand on rice productivity [25], and the consumption and marketing of rice (Ogunleke and Baiyegunhi, 2020; [26]. These studies were carried out across different parts of Nigeria; however, there is a lack of data regarding the productivity differentials between genders among rice farmers in South-West Nigeria. On the other hand, this study looked at the disparity between genders in South-West Nigerian smallholder rice farmers' productivity. A caveat for this study is that the productivity difference between average male and female smallholder rice farmers analysed using the [27,28] decomposition method of the wage gender gap. This study is presumably among the first to analyze the productivity difference between Nigerian smallholder farmers who are male and female using the Oaxaca-Blinder (OB) decomposition approach. This study follows [21,29] in providing a tool for the design of more effective involvements by examining the policy implications of women's role in households and identifying the drivers of the gender gap. Additionally, using an exogenous switching treatment regression approach, we determined how gender affects rice productivity in relation to various forms of discrimination against women and inequalities. Pooled regression with a gender dummy indicator variable was employed in earlier research.

## 2 Review of literature

### 2.1 Agricultural sector in Nigeria

Agriculture as a sector is known to have the potential to boost welfare and employment in general[30]. In a similar vein, the United Nations' Sustainable Development Goals (SDG) place significant emphasis on the role that agriculture plays in improving social well-being. In order to end world hunger, SDG No. 2, "Invest in Agriculture," underlined the significance of raising agricultural productivity in order to boost productivity and support sustainable food systems. SDG No. 1, "No Poverty," highlighted the necessity of reducing poverty through increased agricultural production. Similar to this, it has been proposed that the agricultural sector serve as a means of subsistence, especially for developing economies in order to achieve SDG Goal No. 4 and, ultimately, SDG Goal No. 12, which can be attained through raising sustainable agricultural production.

Over time, the Nigerian government has diversified the economy and promoted inclusive growth by investing in the agricultural sector and implementing various policies and programs. But given the persistent increases in employment and the poverty rate, concerns have been expressed about the initiatives[31]. Among the initiatives are the Green Revolution, Operation Feed the Nation, and the National Poverty Eradication Program. Before the oil boom of the 1970s, Nigeria's primary industry was its agricultural sector. Nigeria's low GDP contribution from agriculture has resulted in a decline in the welfare of low-income earners. Concerns regarding the sustainability of the economy have also been raised by the recent drop in oil prices on the world market. Nigeria's agricultural sector generated nearly 50 % of government revenue, 65 % of GDP, and over 80 % of export earnings in the 1960s. This has become less of an asset to Nigeria's economic expansion over time. Roughly 26.84 % of the GDP was derived from agriculture in 2021 [32]. Agriculture still contributes to output and jobs, despite a recent decline[33].

### 2.2 Gender and agricultural productivity

In developing nations, the topic of gender disparities in farm productivity in subsistence farming has entered the public policy discourse[34,35]. In Africa, where there is a great deal of variation in cultural and religious beliefs, figuring out the productivity level of male and female farmers is important for improving food security [36]. In addition to having a negative impact on women's empowerment in developing nations, the gender gap in the adoption of modern crop varieties and other agricultural technologies has a real cost to society in the form of wasted potential for increased agricultural productivity, food security, and economic growth [37,38]. According to empirical research, maize yields in Malawi, Ghana, and western Kenya could rise by up to 16 %, 17 %, and 19 %, respectively, if female farmers had equal access to improved agricultural inputs like fertilizer and seed as male farmers do[39]. According to Ref. [40]; Ghanaian women farmers produce more rice than their male counterparts. This result is also consistent with the research of [41]; who found that the inefficiency of female rice farmers was caused by barriers to the acquisition of useful inputs. On the other hand [42], demonstrated in a related study that male farmers in Kenya are more productive than female farmers.

[43] found no statistically significant difference between the technical efficiency of male and female farmers in Benin, but they did observe a significant difference in the paddy rice yield of men and women, with a larger yield by male farmers. They clarified that the reason male farmers were more productive than female farmers was because they owned larger land holdings that they used for rice farming. Additionally, studies conducted in developing nations revealed that women's control over income has a bigger positive impact on household members' calorie intake, nutrition, health, and educational attainment than does men's control over income[44].

[45] measured the gender gap in modern maize adoption and looked into how Malawi's Farm Input Subsidy Program (FISP) affected the gap. However, their findings differed from those of earlier studies by Refs. [46,47]; which showed that gender was a significant factor in modern maize adoption even after controlling for characteristics at the individual, household, and community levels. According to their research, the adoption probability of modern maize by female household heads increased by 22.2 % when FISP coupons were used; however, this increase had no effect on the adoption probability of wives in male households.

### 2.3 Conceptual framework

#### 2.3.1 Tests of hypothesis for the study

**Fig. 1:**
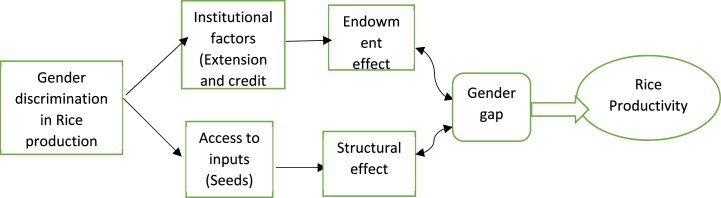
Conceptual framework on gender gaps in rice productivity in Southwest of Nigeria.

In Southwest Nigeria (Fig. 1), the present study focuses on the disparities between men and women's productivity levels with regard to productive resources and institutional elements (land, seeds, loan access, and extension services). It was therefore assumed that there was severe discrimination against men and women in the study area when it came to access to land and equipment. We can test for discrimination on land access directly by comparing the average size of land holdings between the groups of men and women. We compare the mean ages of the male and female nurseries on the day of transplantation in order to test for gender discrimination indirectly. This makes it possible for us to present the delay in transplanting rice seedlings from the nursery to the main fields as proof that discrimination in the use of equipment exists. One of the main theories in this study is that if discrimination on land and equipment didn't exist, there wouldn't be a productivity gap between men and women. To determine whether there is equality of means, the average and marginal productivities of men and women are compared.

#### 2.3.2 Gender decomposition: Oaxaca-Blinder (OB) approach

The productivity difference between the average male and female farmer was broken down into two components using an OB decomposition methodology, in accordance with the traditional approach of the wage gender gap literature of [27,28]. These components are (i) the endowment effect and (ii) the structural effect. While gender disparities in the returns to these factors are linked to the latter, the former is explained by unequal access to production inputs and farmer characteristics. As observed in the majority of other studies, endogeneity in production inputs is an inevitable problem since this method builds upon fundamental OLS regression estimates[48,49].

The literature on gender and union wage gaps had made extensive use of a variety of decomposition techniques. Additionally, they had been used to determine the variables that account for economic growth and changes in inequality[50]. [21] proposed that variations in the returns to extension services, land certification, land extension, and product diversification may contribute to the unexplained portion of smallholder farmers' productivity in Ethiopia when analyzing the breakdown of gender disparities in agricultural productivity in Ethiopia. In order to ascertain how much variations in the levels and returns to productivity determinants account for the overall gender disparity in agricultural productivity, this article employs a decomposition method. The primary goal of the decomposition approach is to partition the total difference between two groups' relevant statistics from a given distribution as expressed in Equation (1):

(1) $$\Delta_{o}^{u}=u\left(Q_{Y_{B}/Z_{B}}\right)-u\left(Q_{Y_{A}/Z_{A}}\right)$$

u(.), the statistic in which *ν* (·) is the statistic of concentration, typically the average, $\left(Q_{Y_{B}/Z_{B}}\right)$ is the cumulative distribution of the latent outcome, $Y_{qi}$, for individuals of group S. For this study, the male and female smallholder rice farmers are the mutually exclusive groups. In constructing counterfactuals, a structural form pointing to the outcome with observed and unobserved individual characteristics ($X_{i}$ and $\varepsilon_{i}$, respectively) is stated. To present a simple assumption of counterfactual treatment, overlapping support, and ignorability, the overall difference, $\Delta_{o}^{u}$, in Equation (2) can be split into two terms after adding and subtracting $u\left(Q_{Y_{A}/Z_{A}}\right)$ from Equation (1):

(2) $$\Delta_{o}^{u}=\underset{\Delta_{s}^{u}}{\underbrace{\left(u\left(Q_{Y_{B}/Z_{B}}\right)-u\left(Q_{Y_{A}^{C}/Z_{B}}\right)\right)}}+\underset{\Delta_{X}^{u}}{\underbrace{\left(u\left(Q_{Y_{A}^{C}/Z_{B}}\right)-u\left(Q_{Y_{A}/Z_{A}}\right)\right)}}$$

where $\Delta_{s}^{u}$ or the “structural effect” represents differences in returns to observable characteristics, and $\Delta_{X}^{u}$ or the “endowment effect” reflects differences in the distribution of noticeable characteristics between both groups. The fundamental mean decomposition employs the technique of [27,28]; enabling the estimation of Equation (2) through the imposition of the subsequent assumptions:

***Additive linearity*** implies that the structural form can be characterized as a linear additively separable function of individuals’ observed and latent features (Equation (3)):

(3) $$Y_{qi}=X_{i}\alpha_{q}+u_{iq}\text{where}q=\{A\text{,}B\}\text{and}u_{iq}=k_{q}\left(\varepsilon_{i}\right)$$

$X_{i}$ is a vector of features and $\alpha_{q}$ is a vector of OLS parameters, estimated separately for each group.

***Zero conditional mean*** indicates that $Ε\left(u_{iq}/X_{i,}Z_{Bi}\right)=0$.

Applying assumptions 1 and 2 to the OB framework is obtained as:

(4) $$\Delta_{o}^{u}=\left(u\left(Q_{Y_{B}/Z_{B}}\right)-u\left(Q_{Y_{A}^{C}/Z_{B}}\right)\right)+\left(u\left(Q_{Y_{A}^{C}/Z_{B}}\right)-u\left(Q_{Y_{A}/Z_{A}}\right)\right)=Ε\left(X_{i,}/Z_{Bi}\right)\left(\alpha_{B}-\alpha_{A}\right)+Ε\left(X_{i,}/Z_{Ai}\right)\left(\alpha_{B}-\alpha_{A}\right)=\underset{\Delta_{S}^{U}}{\underbrace{Ε\left(X_{i,}/Z_{Bi}\right)\left(\alpha_{B}-\alpha^{*}\right)+Ε\left(X_{i,}/Z_{Ai}\right)\left(\alpha^{*}-\alpha_{A}\right)}}+\underset{\Delta_{X}^{U}}{\underbrace{Ε\left(X_{i,}/Z_{Bi}\right)-Ε\left(X_{i,}/Z_{Ai}\right)\alpha^{*}}}$$

where, in the last step, $Ε\left(X_{i,}/Z_{Bi}\right)\alpha^{*}$ and $Ε\left(X_{i,}/Z_{Ai}\right)\alpha^{*}$ are added and subtracted to derive an alternative measure for the structural effect. The structural effect, $\Delta_{s}^{u}$ in equation (4), is thus the sum of two terms: male structural advantage, $Ε\left(X_{i,}/Z_{Bi}\right)\left(\alpha_{B}-\alpha^{*}\right)$, and female structural disadvantage, $Ε\left(X_{i,}/Z_{Ai}\right)\left(\alpha^{*}-\alpha_{A}\right)$. An extensive decomposition evaluating each covariate's contribution to the structural and endowment effects can be estimated, provided the additive linearity assumption is met.

It was believed that the manager's sociodemographic characteristics, labor and non-labor inputs, and farm size all influenced agricultural productivity. These covariates are expressed as inputs per unit of land in the regression analysis of this study, which employs a yield-based methodology[49]. While there may be an endogeneity issue with this specification [48,49], the goal of this study is to determine the degree to which variations in the observed variables account for the gender gap in productivity rather than to infer causality. This information will help guide policy by pointing out potential areas of intervention. Although it was still possible that bias in the estimates could result from other, unobservable terms, a series of robustness checks was carried out to test for this. If the ignorability assumption is false, bias in the estimates could arise.

#### 2.3.3 Exogenous switching treatment effect regression

An exogenous switching treatment effect regression (ESTER) was utilized to estimate the causal effect of gender on rice productivity of various inequalities. It might not be appropriate to evaluate the relationship between gender and rice productivity using a pooled regression, which is a dummy regression in which a binary gender variable is used. This is so because the estimation of a pooled regression model relies on the assumption that the set of covariates affects FHHs and MHHs in the same way (i.e., that both groups' slope coefficients are the same). Gender only has an intercept effect, or a parallel shift effect, which is constant regardless of the values taken by other covariates that determine productivity. This suggests that there is no interaction between the gender variable and other explanatory variables. Refer to the study of[51,52]. for the model and mathematical specification.

## 3 Study area and source of data

The study was conducted in what is known as the South-West geographical zone of Nigeria, which includes the states of Lagos, Ogun, Oyo, Osun, Ondo, and Ekiti. The region, which spans a total land area of roughly 77,818 km^2^, is located between the longitudes 2^31′E and 6^00′E and the latitudes 6^21′N and 8^37′N. The Gulf of Guinea borders it on the south, the Republic of Benin on the west, the Kwara and Kogi states on the north, and the Edo and Delta states on the east. South-West Nigeria experiences tropical weather with distinct wet and dry seasons. The annual rainfall varies from 150 mm to 3000 mm, and the mean temperature is between 21 °C and 34 °C. The trade wind from the Sahara Desert in the northeast is linked to the dry season, while the monsoon wind from the southwest of the Atlantic Ocean is linked to the wet season. The lowland forest extends inland to the Ogun and portions of the Ondo states, while the secondary forest stretches towards the northern boundary by the derived and southern Guinea savannas. The vegetation of South-West Nigeria is composed of freshwater swamp and mangrove forest at the belt[53].

The study employed a multistage sampling technique to choose the participants. In the first phase, three states—Ekiti, Ondo, and Osun—that are all part of the same agro-ecological region were chosen primarily for purposeful cases. Using a typical case purposive sampling technique and taking into account the preponderance of smallholder rice farmers in each state, four local government areas (LGAs) were chosen for the second stage. Five villages were chosen at random from each of the four LGAs for the third stage. In accordance with [54]; the sample size for the research was established using the sample determination formula as outlined by Ref. [55] with a 95 % confidence level and 5 % margin of error. This allowed for the selection of six smallholder rice farmers from each of the five villages that had previously been chosen. 360 respondents were subsequently contacted for study interviews. A well-structured, pre-tested questionnaire on respondents' socioeconomic traits and rice productivity was used to gather data.

## 4 Empirical results and discussions

Table 1 displays the descriptive statistics of the rice farmers who were surveyed. Here, the productivity gap between men and women in terms of farm plot management was broken down using the standard OB decomposition technique into two categories: those that can be explained by differences in the factors influencing productivity and those that cannot. Table 2 displays the outcomes of the group-specific regression for both males and females. The primary factors that determine variations in productivity are age, marital status, level of formal education, and prior farming experience.

**Table 1:** Definitions and summary statistics of variables used in the model.

Variables			
Dependent	Description of variables	Mean	SD
Yield of rice	Ln of rice yield (kg)		
***Independent variables***			
Gender of HH head	1 if household (HH) head is male, 0 if female	0.56	0.50
Age of HH head	Age of HH head (years)	47.28	7.67
Marital status	1 if HH head is married, 0 if other/single/widowed	0.80	0.40
Educational status	Years of education of HH head	6.45	5.70
Household size	Number of HH size	4.66	1.24
Off-farm income	1 = if HH engages in any off-farm activity	0.54	0.50
Farming experience	Years of HH experience in rice production	15.73	5.09
Access to credit	1 if HH has access to credit, 0 if otherwise	0.57	0.50
Farm size	Total land owned by HH, in hectares	7.37	3.04
Access to climate info	1 if HH gets climate change information, 0 if otherwise	0.36	0.48
Access to ext. contacts	1 if HH has access to extension, 0 if otherwise	0.53	0.50
Membership	1 if HH belongs to Farmers' Association	0.54	0.50
Location_Ekiti State	1 if HH is from Ekiti, 0 if otherwise	0.38	0.48
Location_Ondo State	1 if HH is from Ondo, 0 if otherwise	0.38	0.49
Location_Osun State	1 if HH is from Osun, 0 if otherwise	0.35	0.48

**Table 2:** Gender-specific farm productivity models – estimates from the Oaxaca-Blinder (OB) decomposition.

Variable	Male-managed plot		Female-managed plot	
	Coeff.	SE	Coeff.	SE
Socioeconomic characteristics				
Age	0.0066	0.0039^c^	0.0050	**0.0027**^**c**^
Marital status	−0.0293	0.0848	−0.2269	**0.0569**^**a**^
Formal education	−0.2397	0.1347^c^	−0.2995	**0.0857**^**c**^
Household size	0.0024	0.0244	−0.0134	0.0143
Farming experience	−0.0227	0.0159	−0.0186	**0.0104**^**c**^
Farm size allocated to rice farming	0.0874	0.0111^a^	0.1189	**0.0073**^**a**^
Engagement in non-farm activities	0.3589	0.4288	−0.0352	0.1171
***Institutional characteristics***				
Access to extension services	−0.0190	0.2206	0.0112	0.0955
Access to market information	0.0229	0.0828	−0.0704	0.0461
Membership of farmer-based organization (FBO)	−0.2562	0.3778	−0.0019	0.1144
Access to credit facility	0.0754	0.0786	0.0543	0.0454
Constant	8.5950	0.3841	9.0401	0.2926
***Number of observations***	159		201	
*F (11, 147/189); Prob > F*	0.0000		0.0000	
*R-squared*	0.4254		0.7110	
*Adjusted-squared*	0.3824		0.6942	

Coeff. and SE denote coefficient and standard error, respectively. a and c denote significance levels at 1 % and 10 %, respectively.

### 4.1 Descriptive statistics of socioeconomic variables

The average age and number of years of education for household heads are 47 and six, respectively, according to Table 1's results. About 53 % of the respondents said they have communication with extension agents regarding access to extensions. A significant factor in agricultural productivity is credit availability, yet only roughly 57 % of smallholder rice farmers have access to credit. Nonetheless, there are pronounced differences in terms of information availability; for instance, roughly 36 % of farmers who have implemented a strategy at all have access to data on agricultural output. The study area's farmers have an average of fifteen years of farming experience. The outcome is consistent with the hypothesis put forth by Ref. [56]; according to which farmers' ability to allocate resources effectively and their ability to perceive and respond to economic conditions are directly correlated with their endowment of human capital.

### 4.2 Empirical discussions

Plot managers of both genders benefit from increased age in terms of productivity. For men, however, age has a slightly higher rate of return than for women. This may be explained by women's biological makeup and roles, as well as the laborious nature of their household administration duties. The aforementioned biological roles and makeup refer to the fact that women are inherently weaker than men because they give birth to and nurse their children. Furthermore, women in African society are responsible for all household chores and farming, which may weaken them. As a result, as people get older, their ability to put in the necessary effort to produce more decreases[57,58]. Additionally, a few of these elderly women might be widowed, which would give them less financial clout and support than their male counterparts to buy the agricultural inputs needed for increased productivity.

For female managers, marital status has a substantial negative impact, but it has no effect on male managers. Therefore, women who oversee their own farm plots have a lower private rate of return based on their marital status. According to Refs. [59,60]; and [21]; women who are single or who have recently divorced or become widowed are typically the heads of households headed by women. The role of women managers in rural farming communities in Nigeria is contingent upon their social status. Women who have been widowed or divorced typically have less access to resources. Compared to their married counterparts, they are noticeably less productive due to a restriction on their access to resources[21,[61], [62], [63]].

Since education is essential to farmers' adoption of new technology, it should enhance their capacity to comprehend the advantages of new technology (Feder et al., 1985). Remarkably, the longer a plot manager has attended formal schooling, the less productive they will be—male or female. Insufficient knowledge or inadequate field training to address low agricultural productivity in SSA generally, and Nigeria specifically, may be the cause of the negative effect of education on productivity. Similarly, farming experience shows that both male and female plot managers have negative returns. Lastly, there is a strong and positive correlation between the size of the farm used for rice production and the productivity of both sexes. This finding contradicts data from the literature that shows a negative correlation between farm size and productivity[21,[64], [65], [66], [67], [68], [69]]. For women, the returns are higher than for men, though. This happens because men typically oversee larger land areas than women do, which may lead to diminishing marginal returns to productivity and, consequently, lower private rates of return[62].

### 4.3 Oaxaca-Blinder (OB) mean decomposition

The [27,28] decomposition method is the basis for the mean difference in farm productivity between male and female, as was previously mentioned. The average forecasts for men and women, as well as the discrepancy between the two, are shown in Table 3 of the decomposition output. The productivity mean for men and women in the Table is 9.36 and 9.08 respectively, resulting in a noteworthy 0.29 difference in productivity. This implies that there is an unequal farming productivity situation for men and women. Because of this, women are around 29 % less productive than men. The size of this discrepancy is comparable to that found in other African nations [70]. study in Malawi found a gender gap of 25.4 % [21]; found a gender difference of approximately 23.4 % in Ethiopia; and [71] found a gender productivity differential of approximately 18.3 % in Niger.

**Table 3:** Oaxaca-Blinder (OB) decomposition estimates.

Log productivity	Coefficient	Standard error
Mean value for male	9.364^a^	0.037
Mean value for female	9.078^a^	0.319
Mean difference	0.286^a^	0.049
***Decomposition***		
Endowments	0.043	0.0424
Coefficient	0.279^a^	0.038
Interaction	0.488^b^	0.023

a *and*^b^*denote significance level at 1 % and 5 %, respectively*.

Furthermore, a recent study on gender productivity differences across three African nations by Ref. [72] discovered 18.6 %, 27.4 %, and 30.6 % productivity gaps for Nigeria, Tanzania, and Uganda, respectively. If women have similar traits to men, the endowment effects show the mean increase in women's productivity. The endowment factor indicates that women's productivity would have been 0.043 (4.3 %) higher if they had the same productivity characteristics as men. This increase suggests that only 15 % of the productivity gap can be attributed to variations in endowment. Therefore, even at a 10 % level of significance, it is not surprising that the endowment factor is not significant. When female characteristics are combined with male coefficients, the resultant change in female productivity is quantified by the structural effect, or coefficient factor. Equal opportunities for men and women would therefore result in a significant 28 % increase in women's productivity.

Roughly 98 % of the productivity gap can be explained by the structural effect. This study's results are consistent with those of [21]; but they deviate from earlier research[73]; Kinkingninhoun-Mêdagbé et al., 2010 [74,75]; that found that variations in production characteristics account for the majority of the gender disparities in farm productivity. The interaction term is a measure of the simultaneous effects of endowment and coefficient differences. The study strengthens the analysis by identifying factors contributing to the endowment and structural effects that result from breaking down the farm productivity gap. As previously mentioned, a thorough breakdown of the factors influencing endowment and structural effects can be estimated provided the additive linearity assumption is met. The outcomes of the thorough breakdown of the identical variables listed in Table 1 are shown in Table 4.

**Table 4:** Detailed decomposition of endowment and structural effects.

Variables	Endowment effects		Structural effects	
	Coeff.	SE	Coeff.	SE
Socioeconomic characteristics				
Age	0.0029	0.0018	−0.0770	0.2223
Marital status	−0.0221	0.0058^a^	−0.1479	0.0764^b^
Formal education	−0.0152	0.0034^a^	−0.0339	0.0904
Household size	−0.0012	0.0014	−0.0731	0.1305
Farming experience	0.0078	0.0056	0.0747	0.3447
Farm size allocated to rice farming	−0.0139	0.0005^a^	0.2346	0.0989^b^
Engagement in non-farm activities	−0.0018	0.0033	−0.2032	0.2292
***Institutional characteristics***				
Access to extension services	0.0003	0.0002	0.0150	0.1194
Access to market information	−0.0046	0.0012^a^	−0.0305	0.0310
Membership of FBO	0.0005	0.0035	0.1327	0.2061
Access to credit facility	0.0052	0.0037	−0.0084	0.0360

*Coeff. and SE denote coefficient and standard error, respectively.*^*a*^*and*^*b*^*denote significance levels at 1 % and 5 %, respectively*.

The detailed decomposition's results show that endowment effects are influenced by a number of factors, including farm size, education level, marital status, and access to market data. According to Table 1's descriptive statistics, 75 % of the women and 85 % of the men in the data sample are married. Consequently, compared to the approximate 15 % of men with similar statuses, approximately 25 % of women are divorced, widowed, and single. The amount of farmland devoted to rice production is a significant additional factor in the endowment effect. There is no discernible difference between the amount of land that men and women devote to rice production, according to Table 4's results. This runs counter to numerous examples in the literature that indicate women's access to land is severely restricted, especially in SSA[21,[76], [77], [78]].

Farm size and marital status are the primary factors that contribute to the structural effects. Since the coefficients cannot be interpreted causally, the study attempts to be cautious in its interpretation of the determinants of the structural effects[48,[49], [79]]. As a result, the findings can only be suggested as guidelines for future research and policy at the farm level.

Table 5 presents the results of an analysis using the ESTER model to determine how the gender of the household head affects rice yield for both MHHs and FHHs. The outcome demonstrates that the rice yield of FHHs would have decreased by 25.41 kg/ha (a 1.02 % reduction) if they had been given the same returns to the observed characteristics of MHHs. This difference is significant at the 1 % level. It would be incorrect to conclude, despite the statistical significance, that FHHs have a rice yield advantage based solely on their characteristics. This is because the differences in rice yield are too slight to suggest that gender significantly influences rice yield. Therefore, the findings imply that the FHHs are not at a lower yield than the MHHs. This is consistent with the findings of Ali et al. (2016), who found that in rural Uganda, female-managed plots had a net endowment advantage of 12.9 % despite men having greater access to resources and inputs. Additionally, the findings demonstrate that rice yield has a base level effect on MHHs of 1.02 % (significant to the 1 % level). This suggests that if FHHs and MHHs had used the same amount of resources, FHHs' rice yield would have decreased by 1 %.

**Table 5:** Gender differential in rice yield-exogenous switching regression model.

Outcome	Female HHs	Male HHs	Returns effect	% Gain
Rice yield for Female HH	1639.176 (5.485)	1641.343 (7.528)	−2.16	1.00
Rice yield for Male HH	1664.591 (5.329)	1691.238 (6.920)	−26.64***	1.02
Level effects	−25.415***	−49.895***		
% Gain	1.02	1.03		

Standard errors are in parenthesis. *** denotes significance level at 1 %.

## 5 Conclusion and policy recommendations

This study used data from the Nigerian states of Osun and Ogun to examine differences in farm productivity between male and female rice farm managers. The study adds to the body of knowledge on gender studies in Sub-Saharan Africa. The results show a gender productivity gap of 0.29 using the OB gender decomposition model, indicating that female plot managers are 29 % less productive than their male counterparts. The structural effect, or the portion of the gender differential due to returns of the same characteristics, accounts for approximately 98 % of the gap in productivity between men and women, while the endowment effect, which measures the proportion of gender productivity gaps attributable to differences in observable characteristics between men and women, accounts for about 15 % of the gap. This outcome is consistent with other SSA studies that computed productivity and wage disparities between men and women using the same framework. Thus, this repeating pattern points to an SSA structure where women's productivity is lower than men's. The group-specific regression's findings show that the productivity gaps between men and women are mostly caused by variations in the private rates of returns to variables like age, marital status, length of formal education, and farming experience.

Sequel to the endowment effects, the most important covariates that explain productivity differences between men and women are married status, education level, farm size, and access to market information. Since the coefficient could not be interpreted as causality, the results from the structural effects section of the detailed decomposition prevented this study from offering any additional explanation. The factors influencing productivity do, however, have some policy implications at the farm level. Encouraging women to reach their full potential would have resulted in measurable gains from closing the gender gap in agricultural productivity. Given that Nigeria's economic growth is primarily determined by agriculture, understanding the productivity gaps and the factors that contribute to them is essential to developing policy interventions that support women's empowerment. In order to account for unobserved specific heterogeneity (such as social, cultural, and political barriers that are not observed by researchers), future analysis using repeated observations (or panel data) may be required to investigate the relationship between gender and productivity. This will allow us to determine whether the MHH-FHH food security gap persists over time. A panel data analysis might help reveal whether or not gender-specific norms change over time if, as our results imply, they contribute to the difference in productivity. Further studies on the relationships between various FHHs and food security may also be essential for identifying FHHs with low productivity and recommending suitable policy measures.

## Availability of data and materials

The data that support the findings of this study can be obtained from the authors upon request.

## Ethics approval and consent to participate

The study received an ethical clearance and each participant signed a consent form.

## Funding

This research did not receive any specific grant from funding agencies in the public, commercial, or not-for-profit sectors.

## Data availability

Data will be made available on request.

## CRediT authorship contribution statement

**Temitope O. Ojo:** Writing – original draft, Methodology, Formal analysis, Data curation, Conceptualization. **Lloyd J.S. Baiyegunhi:** Writing – review & editing, Supervision.

## Declaration of competing interest

The authors declare that they have no competing interests.

## Figures and Tables

**Table:** 

qtyproducedhakg	Coef.	St.Err.	t-value	p-value	[95 % Conf	Interval]	Sig
age	−0.282	1.206	−0.23	0.815	−2.661	2.097	
maritalstatus	−139.559	24.369	−5.73	0.000	−187.634	−91.485	***
yearsspentinschool	−15.573	1.438	−10.83	0.000	−18.411	−12.735	***
hhsize	4.033	6.366	0.63	0.527	−8.526	16.591	
offfarminco	−13.183	52.866	−0.25	0.803	−117.473	91.108	
riceexperience	0.990	2.260	0.44	0.662	−3.469	5.449	
obtainloan	32.765	20.014	1.64	0.103	−6.718	72.247	
farmsize	−0.876	3.334	−0.26	0.793	−7.454	5.702	
informaccess	−15.830	20.433	−0.78	0.439	−56.139	24.480	
acesstoext	10.800	49.659	0.22	0.828	−87.164	108.764	
membership	15.538	48.252	0.32	0.748	−79.650	110.726	
Annualtemp	−5.447	176.818	−0.03	0.975	−354.261	343.367	
annulppt	0.175	0.515	0.34	0.735	−0.841	1.191	
Constant	1976.569	4903.368	0.40	0.687	−7696.457	11649.594	

Mean dependent var	1641.343	SD dependent var	151.247
R-squared	0.498	Number of obs	201.000
F-test	14.269	Prob > F	0.000
Akaike crit. (AIC)	2476.504	Bayesian crit. (BIC)	2522.750

***p < 0.01, **p < 0.05, *p < 0.1.

**Table:** 

qtyproducedhakg	Coef.	St.Err.	t-value	p-value	[95 % Conf	Interval]	Sig
age	0.056	1.298	0.04	0.966	−2.510	2.622	
maritalstatus	−145.232	25.996	−5.59	0.000	−196.611	−93.852	***
yearsspentinschool	−8.871	2.520	−3.52	0.001	−13.852	−3.891	***
hhsize	−0.937	8.116	−0.12	0.908	−16.978	15.104	
offfarminco	55.083	176.324	0.31	0.755	−293.414	403.579	
riceexperience	−3.813	2.325	−1.64	0.103	−8.409	0.783	
obtainloan	7.362	26.362	0.28	0.780	−44.742	59.465	
farmsize	6.683	3.547	1.88	0.062	−0.327	13.694	*
informaccess	26.528	28.416	0.93	0.352	−29.635	82.690	
acesstoext	−11.535	125.954	−0.09	0.927	−260.478	237.407	
membership	−46.796	127.890	−0.37	0.715	−299.566	205.975	
Annualtemp	39.428	205.410	0.19	0.848	−366.555	445.412	
annulppt	−0.001	0.634	−0.00	0.999	−1.254	1.252	
Constant	734.440	5693.763	0.13	0.898	−10500.000	11987.932	

Mean dependent var	1664.591	SD dependent var	134.267
R-squared	0.251	Number of obs	159.000
F-test	3.728	Prob > F	0.000
Akaike crit. (AIC)	1990.510	Bayesian crit. (BIC)	2033.475

***p < 0.01, **p < 0.05, *p < 0.1.
